# Identification of MYB Transcription Factor, a Regulator Related to Hydrolysable Tannin Synthesis in *Canarium album* L., and Functional Analysis of CaMYBR04

**DOI:** 10.3390/plants13131837

**Published:** 2024-07-04

**Authors:** Qinghua Ye, Huiquan Wang, Zhehui Lin, Qian Xie, Wei Wang, Qingxi Chen

**Affiliations:** 1 Department of Horticulture and Landscape Architecture, Fujian Vocational College of Agriculture, Fuzhou 350303, China; tinayqh@163.com (Q.Y.);; 2 College of Horticulture, Fujian Agriculture and Forestry University, Fuzhou 350002, China

**Keywords:** hydrolysable tannins, polyphenols, MYB, transcription factor

## Abstract

Hydrolysed tannins (HTs) are polyphenols, which are related to the astringency, flavour, colour, stability, medicinal value and other characteristics of many fruits and vegetables. The biosynthetic mechanism of the majority of HTs remains unknown, and many biosynthetic pathways of HTs are speculative conclusions that have not been confirmed. The fruit of *Canarium album* L. (Chinese olive), which is notable for its pharmacological and edible properties, is rich in HTs. The fruit has a distinctive bitter and astringent taste when initially consumed, which mellows to a sweet sensation upon chewing. HTs serve as the primary material basis for the formation of the Chinese olive fruit’s astringent quality and pharmacological effects. In this study, the fruit of *C. album* Changying was utilised as the research material. The objective of this study was to provide a theoretical basis for the quality control of Chinese olive fruit and the application and development of its medicinal value. In addition, the study aimed to identify and screen related MYB transcription factors involved in the synthesis of HTs in the fruit and to clarify the mechanism of MYBs in the process of synthesis and regulation of HTs in Chinese olive fruit. The principal findings were as follows. A total of 83 differentially expressed Chinese olive MYB transcription factors (CaMYBs) were identified, including 54 1R-MYBs (MYB-related), 25 2R-MYBs (R2R3-MYBs), 3 3R-MYBs, and 1 4R-MYB. Through trend analysis and correlation analysis, it was found that *CaMYBR04* (*Isoform0032534*) exhibited a significantly higher expression (FPKM) than the other CaMYBs. The full-length cDNA sequence of *CaMYBR04* was cloned and transformed into strawberry. The results demonstrated that CaMYBR04 significantly enhanced the fruit’s hydrolysable tannin content. Consequently, this study elucidated the function of CaMYBR04, a regulator of the Chinese olive fruit hydrolysable tannin synthesis pathway, and established a theoretical foundation for the synthesis and regulation of fruit HTs.

## 1. Introduction

*Canarium album* L. (Chinese olive), is an evergreen fruit tree belonging to the *Canarium* genus in the Burseraceae family [1]. The tree is tall, with a pronounced trunk, and the plant is resistant to poor soils, including saline and rocky slopes [2]. It is native to China, found in southeastern Fujian, Guangdong, Guangxi, Hainan, and Sichuan, and has been introduced to other tropical and subtropical regions of Asia, including Vietnam, Malaysia, and Japan [2,3]. The Chinese olive is similar to the European Mediterranean olive (*Olea europaea* L.) in that it is a pike-shaped drupe with a strongly bitter flesh. The endocarp is a highly lignified kernel containing seed kernels, which are rich in nutrients [4]. In contrast to *Olea europaea*, which is primarily utilized as a source of olive oil extraction, the Chinese olive is relatively low in oil content and is a tropical and subtropical fruit used for both medicinal and food purposes. Additionally, it can be processed into various products, including beverages, candies, and preserves [5,6]. Mogana and Wiart [7] and Xiang et al. [8] conducted reviews of the potential pharmacological activities and phytochemical composition of extracts from Chinese olive fruits, leaves, bark, and roots. The phytochemical analyses demonstrated that Chinese olives are rich in phenolic compounds, including flavonoids, phenolic acids, and tannins [9,10]. The studies by Chang Qiang [11] have revealed that the phenolic compounds in Chinese olive fruit are primarily gallic acid (GA) derivatives and ellagitannins, with the highest content of 3-O-galloylquinic acid and geraniin. Furthermore, the total hydrolysable tannin (HT) content can reach 56% of the total polyphenol content, which is considerably higher than that of berries that have been identified as having a higher content of hydrolysable tannins (HTs) (2.3 to 13 times) [12,13].

HTs are a large group of phenolic compounds. Phenolic compounds are widely distributed in all plant tissues and play an important role in fruit colour, flavour, quality, and astringency. The majority of phenolic compounds include a variety of by-products produced by the shikimic acid pathway, the phenylpropane pathway, and the flavonoid pathway, ranging from simple structures with aromatic rings to the complex structures of polyphenolic substances, including HTs, condensed tannins, lignans, and other polyphenolic substances [14]. Transcription factors play an important regulatory role in the biosynthesis and metabolism of many phenolic substances, and more studies have focused on compounds such as flavonoids, anthocyanosides, proanthocyanidins, and lignans. Several transcription factors that regulate proanthocyanidin biosynthesis and accumulation have been identified in Arabidopsis, such as TT1 (zinc-finger), TT2 (MYB), TT8 (bHLH), TT16 (MADS), TTG1 (WD40), TTG2 (WRKY), and FUSCA3 (ABI3/VP1) [15].

The MYB transcription factor family represents the largest family of transcription factors involved in regulating gene transcription in plants. MYB transcription factors have been shown to regulate the biosynthesis of lignans, anthocyanins, proanthocyanidins, and other flavonoids in the flavonoid biosynthesis pathway. Their function has been identified and characterised in a variety of plants, as evidenced by the numerous studies that have been conducted on this topic [16,17]. The MYB family can be divided into four subfamilies: the first subfamily is 1R-MYB, also known as MYB-related; the second is 2R-MYB, also known as R2R3-MYB; the third is 3R-MYB, R1R2R3-MYB; and the fourth is 4R-MYB, which is the smallest class [18]. Recent studies have demonstrated that MYB transcription factors play a pivotal role in regulating the biosynthesis of plant secondary metabolites, including flavonoids and various phenolics [19]. MYB also plays an important role in the regulation of tannins, which affect the astringent flavour of fruits, and has been studied in grapes, persimmons and peaches [20]. However, the role of MYB transcription factors in the regulation of HTs in Chinese olive fruit remains to be elucidated. In this study, transcriptome analysis of Chinese olives at different developmental stages revealed that the MYB transcription factor family exhibited the most differentially expressed members, including 151 members, accounting for 8.65% of the total. To identify the key MYB transcription factors that regulate HT metabolism in Chinese olive fruit, this study further identified and analysed the differentially expressed MYB transcription factors and explored the potential functions of the key Chinese olive MYB genes through trend analysis and transient overexpression in strawberry. This will provide an important theoretical basis for the further study of the mechanism of Chinese olive HT metabolism.

## 2. Results

### 2.1. Identification and Analysis of the Chinese Olive MYB Transcription Factor Family

#### 2.1.1. Identification and Physicochemical Properties of CaMYB

A total of 152 potential MYB transcription factors were identified based on PicBio SMRT-seq and RNA-seq data (PRJNA749395). Subsequently, 83 differentially expressed Chinese olive MYB transcription factor protein sequences (CaMYB) were identified following structural domain validation by HMMER and the removal of redundant sequences (Table S1). The physicochemical properties of the CaMYB protein sequences were analysed, which revealed that the lengths of the CaMYB members ranged from 71 amino acids (Isoform0122550) to 1766 amino acids (Isoform0109568), with molecular weights from 8.27 kDa (Isoform0005912) to 193.11 kDa (Isoform0109568) and a pI from 4.40 (Isoform0005912) to 11.77 (Isoform0122550). The mean amino acid length was 444.51, the mean molecular weight was 49.21 kDa, and the mean pI was 7.32. Subcellular localisation predictions indicated that all MYB transcription factors were localised to the nucleus, with four MYBs additionally localised to the mitochondria (Isoform0004472, Isoform0013930, Isoform0018829, and Isoform0122550) and one MYB (Isoform0039629) localised to chloroplasts in addition to the nucleus. Further detailed information, please refer to Table S1.

#### 2.1.2. CaMYB Classification, Phylogeny and Conserved Motif Analysis

**Figure 1:**
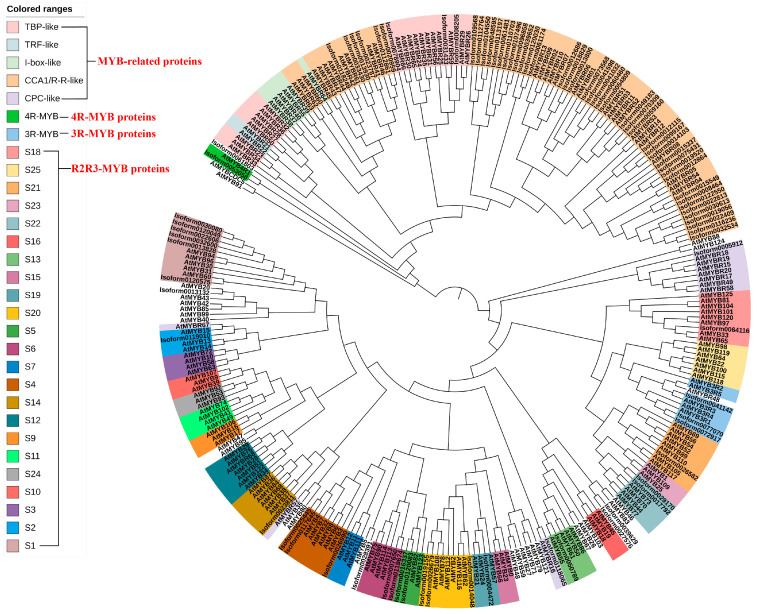
Evolutionary relationship between the Chinese olive differentially expressed MYB transcription factor and Arabidopsis thaliana MYB members. All proteins were classified into four major subfamilies; MYB-related contained five groups: TBP-like, TRF-like, I-box-like, CCA1/R-R-like, and CPC-like; R2R3-MYB contained 23 branches, and 3R-MYB and 4R-MYB transcription factors were also identified.

**Figure 2:**
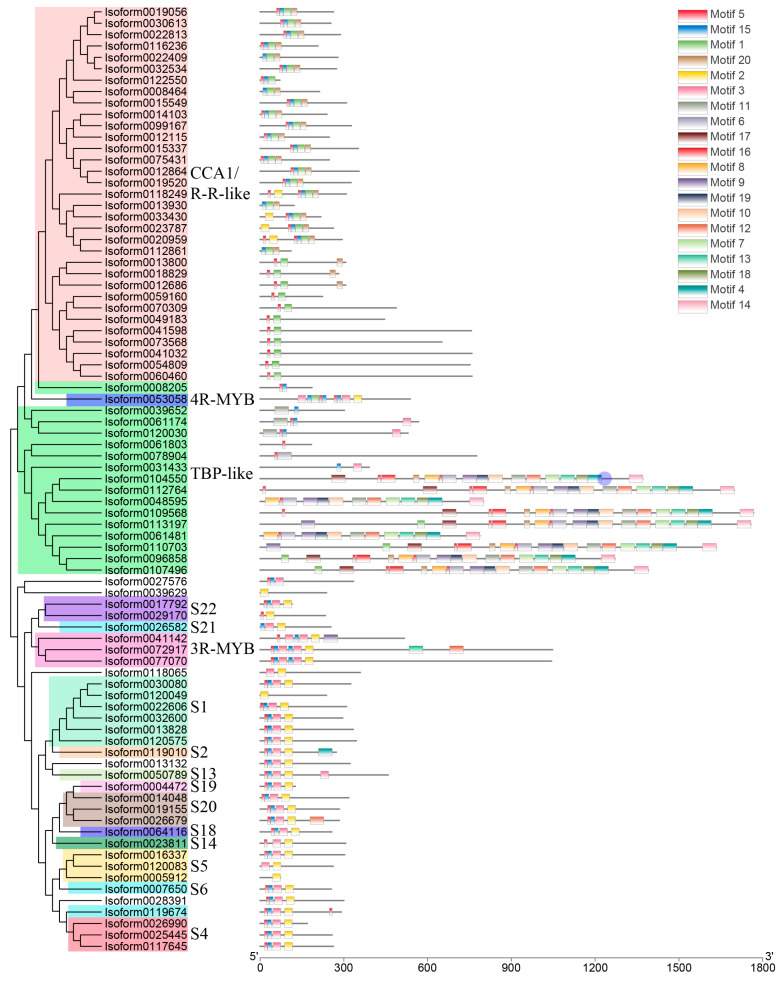
Distribution of conserved motifs of Chinese olive MYB transcription factors based on evolutionary relationships. The phylogenetic tree and different subfamilies of 83 CaMYBs are shown on the left. The 20 conserved motifs of each member are shown on the right, and the sequence information of each motif is shown in Figure S1.

Following Pfam annotation, it was determined that Chinese olive MYB transcription factors contained one to four repetitive sequences. Additionally, Chinese olive MYB transcription factors exhibited differential expression in four subfamilies, comprising 54 1R-MYBs (MYB-related), 25 2R-MYBs (R2R3-MYBs), 3 3R-MYBs, and 1 4R-MYB. A total of 83 differential CaMYBs were identified. A phylogenetic tree was constructed by combining Chinese olive and Arabidopsis MYB members to clarify the evolutionary information of Chinese olive MYB transcription factors, and the results are shown in Figure 1. To investigate the structural diversity of Chinese olive differentially expressed MYB transcription factor proteins, the distribution of conserved motifs based on gene evolutionary relationships was carried out by MEME online software (http://meme-suite.org/, accessed on 1 April 2020). The results are presented in Figure 2. A total of 83 CaMYBs were identified that recognise 20 conserved motifs each, with different numbers of motifs for different phylogenetic taxa.

A phylogenetic tree and conserved motif analysis (Figure 1 and Figure 2) revealed that Chinese olive MYB transcription factor proteins were classified into four major subfamilies, consistent with Pfam annotation. Arabidopsis MYB-related proteins are classified into five groups: TBP-like, TRF-like, I-box-like, CCA1/R-R-like, and CPC-like. In Chinese olive, only two subfamilies, CCA1/R-R-like and TBP-like, were differentially expressed, with 33 and 16 members, respectively. Arabidopsis R2R3-MYB can be classified into 23 categories from S1 to 25 [18], whereas in Chinese olive, only 12 categories were observed: S1, S2, S4, S5, S6, S13, S14, S18, S19, S20, S21, and S22. Studies [21] have demonstrated that MYB-related transcription factors play diverse roles in plant growth and development, including epidermal cell developmental patterns, leaf senescence, circadian regulation, and stress responses. 2R-MYB transcription factors are involved in the regulation of secondary metabolism and plant-specific developmental processes. 3R-MYB regulates various aspects of the cellular developmental cycle. In contrast to the other subfamilies of MYB transcription factors, the understanding of 4R-MYB functions is less well understood than those of other MYB transcription factor subfamilies. Phylogenetic tree analysis provided a more comprehensive understanding of the functional differences of the Chinese olive MYB gene family during evolution. The results showed that the Chinese olive fruit differential transcription factor MYB was predominantly distributed within the 1R-MYB (MYB-related) and 2R-MYB (R2R3-MYB) subfamilies, with 54 and 25 members, respectively. These MYB transcription factors may play pivotal regulatory roles in Chinese olive fruit.

#### 2.1.3. Expression Patterns of Chinese Olive MYBs Transcription Factors and Selection of Key MYB

**Figure 3:**
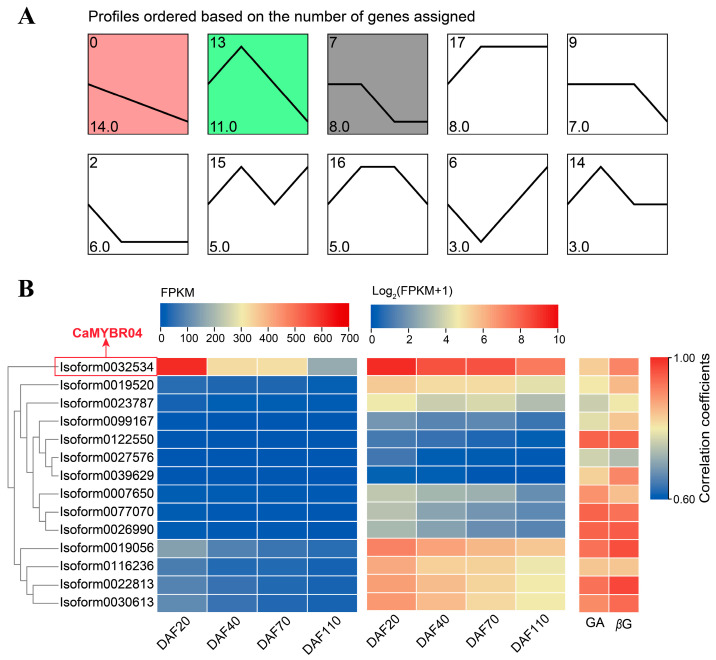
Trend analysis and expression patterns of differentially expressed MYB transcription factors in Chinese olive. (**A**) Trend analysis of differentially expressed MYB transcription factors in *C. album*; the horizontal coordinate illustrates the developmental stages of the *C. album* fruit, while the ordinate depicts the number of genes that align with this trend. The clustered profiles with *p*-value ≤ 0.05 were considered as significant profiles, which were colored. (**B**) Heatmap of gene expression from Profile 0 in the trend analysis, expressed as FPKM and log_2_ (FPKM + 1), respectively. Correlation coefficients between gene expression levels and Chinese olive GA and *β*G content are shown. DAF is an abbreviation for days after flowering.

A trend analysis of the expression patterns of the differentially expressed CaMYB transcription factors was conducted, and the results are presented in Figure 3A. The largest number of genes was observed in profiles 0, 13, and 7, which were significant at the 0.05 level (*p* < 0.05). The most significant profile was profile 0 (*p* = 8.4 × 10^−7^), which exhibited a downregulation trend in gene expression. Profile 0 comprised 14 MYB transcription factors, which exhibited a consistent trend with the Chinese olive fruit GA and *β*-glucogallin (*β*G) content, as evidenced by a high correlation coefficient (Figure 3B). The expression heat map (Figure 3B) indicates that Isoform0032534 exhibited the highest expression FPKM, which was highly significantly higher than the other CaMYB transcription factors. Furthermore, the correlations between this transcription factor and GA and *β*G content were 0.832 and 0.909 (*p* < 0.001), respectively. These findings suggest that this MYB transcription factor may potentially regulate the synthesis of *β*G in Chinese olive fruit. Figure 1 illustrates the phylogenetic relationship between Isoform0032534 and other members of the 1R-MYB subfamily. Isoform0032534 is the closest relative to Arabidopsis AtMYBR04, and thus it was designated CaMYBR04. Subsequent investigations were conducted to elucidate the gene function of CaMYBR04.

### 2.2. Cloning and Analysis of CaMYBR04

**Figure 4:**
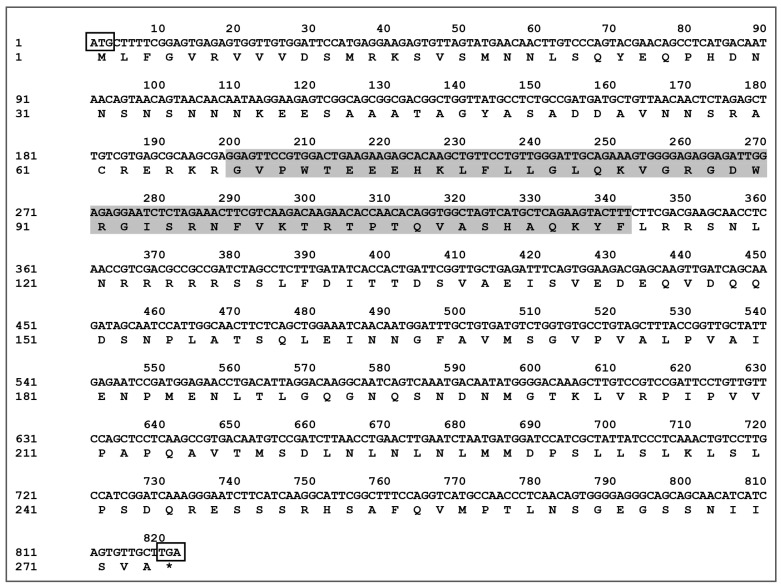
Sequence of the *CaMYBR04* transcription factor CDS and the encoded amino acid sequence. ATG surrounded by a rectangle: start codon; TGA surrounded by a rectangle: stop codon, indicated by “*”. The shaded area indicates the Myb_DNA-binding conserved structural domain.

The CDS sequence of *CaMYBR04* was cloned and subsequently sequenced and spliced. This process yielded the full-length transcription factor *CaMYBR04*, which was determined to be 819 bp in length and encode 273 amino acids. These findings are presented in Figure 4. The predicted molecular weight of the encoded protein was determined to be 30.069 kDa, with an isoelectric point of 6.93. Additionally, the encoded protein was found to contain the Myb_DNA-binding conserved structural domain (PF00249.31).

The CDS fragment of *CaMYBR04* was ligated into the Gateway vector pENTR/D-TOPO using Gateway technology, followed by homologous substitution with the overexpression vector pK7FWG2, and transfected into *E. coli* receptor cells. A positive polymerase chain reaction (PCR) was conducted on individual colonies, and the bacterial fluids exhibiting the correct bands were submitted for sequencing (Figure S2). Following the successful sequencing of the positive clone, the plasmid was extracted in order to obtain recombinant plasmid pK7FWG2-*CaMYBR04*. Subsequently, the transformed competent cells of Agrobacterium tumefaciens were subjected to a second verification of the bacterial liquid PCR. The correct positive clone was preserved in the bacteria and stored at −80 °C.

### 2.3. Subcellular Localisation of CaMYBR04

**Figure 5:**
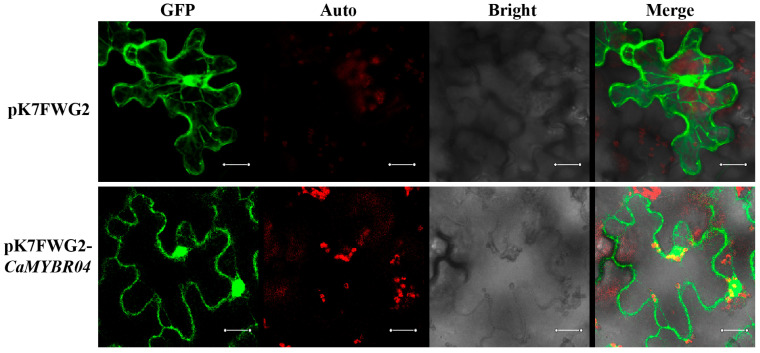
Subcellular localisation of *CaMYBR04.* The laser channels are as follows: GFP-excited fluorescence, chloroplast autofluorescence, bright field map, and merge. *n* = 3 technical replicates for each sample; *n* = 3 biological replicates. The scale bar is 20 μm.

The activated fusion vector pK7FWG2-*CaMYBR04*, which contains the GFP protein tag, and an Agrobacterium tumefaciens empty of the pK7FWG2 plasmid were transiently transformed into the leaf epidermal cells of Ben’s tobacco, respectively. The fluorescence signals of the tobacco leaves were observed with a laser confocal microscope after 36 h of culture. As shown in Figure 5, green GFP fluorescence signals were observed in both the nucleus and the cell membrane. This indicated that *CaMYBR04* was localised in the nucleus and the cell membrane, as previously known (Table S1).

### 2.4. Verification of Gene Function by Transient Overexpression of CaMYBR04 in Strawberry

#### 2.4.1. Positive Identification of Strawberry Transformed with CaMYBR04

To investigate the expression and function of *CaMYBR04* in fruits, the diploid forest strawberry Ruegen was selected as a transient transformation material. Agrobacterium was activated and injected separately into the small green strawberry Ruegen fruits, containing either the overexpression vector pK7FWG2-*CaMYBR04* (CaMYBR04) or the empty pK7FWG2 plasmid (EV). Each transiently transformed strawberry fruit was subjected to PCR analysis using vector-specific primers, as illustrated in Figure S2: Overexpression vector pK7FWG2-*CaMYBR04* positive clone detection. Figure S3: The PCR product bands (615 bp) were correctly positive clones, thereby confirming the successful transfection of *CaMYBR04* into strawberry fruits.

#### 2.4.2. Transient Expression of CaMYBR04 in Strawberry

**Figure 6:**
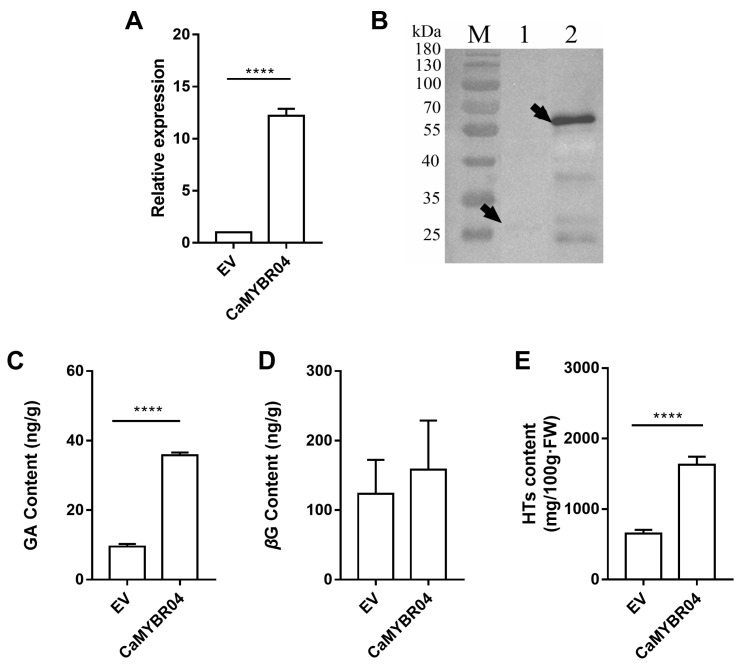
Transient expression of *CaMYBR04* in strawberry fruits. (**A**) shows the relative gene expression of *CaMYBR04* transient strawberry. (**B**) shows the Western blot assay of *CaMYBR04* transient strawberry. The vector pK7FWG2 was GFP-tagged (26.893 kDa) and the overexpression vector was fused with CaMYBR04 protein (30.069 kDa) to obtain a 56.962 kDa fusion protein. The arrows indicate the expressed target proteins. M: marker, Lane 1: anti-GFP, lane 2: CaMYBR04-GFP, EV: empty vector. (**C**–**E**) show the transient strawberry GA (gallic acid), *β*G (*β*-glucogallin), and HT (hydrolysable tannin) content. The symbol “****” indicates a statistically significant difference at the 0.0001 level. The experiment was conducted in three biological replicates per treatment with at least 10 fruits per biological replicate (*n* = 3 technical replicates for each sample; *n* = 3 biological replicates).

After positive identification, the strawberry samples were subjected to subsequent RT-qPCR assay, Western blot analysis and metabolite determination. The results are shown in Figure 6. The gene expression levels following CaMYBR04 transformation were found to be significantly higher than those observed in the EV control (*p* < 0.0001) (Figure 6A). The overexpression vector pK7FWG2 was GFP-tagged (26.893 kDa) and fused with CaMYBR04 protein (30.069 kDa) to obtain a 56.962 kDa fusion protein. The Western blot results (Figure 6B) demonstrated the presence of the target protein bands in strawberry fruits overexpressing *CaMYBR04* (lane 2), indicating that *CaMYBR04* was successfully expressed in strawberry fruits. The GA, *β*G, and HT contents of transiently transformed strawberry fruits were further determined and compared with EV. The results demonstrated that *CaMYBR04* transformation significantly increased the GA content of strawberry fruits and increased the *β*G content, although the difference was not significant. Additionally, the transformation promoted the synthesis of hydrolysed tannins by *β*G, resulting in a highly significant increase in the content of transformed HTs compared with EV (Figure 6C–E). Consequently, the CaMYBR04 fusion protein was successfully expressed in strawberry fruit, thereby accelerating the further transformation of *β*G to increase the hydrolysed tannin content of the fruit.

## 3. Discussion

### 3.1. Analysis and Screening of Differentially Expressed MYB Transcription Factors in Chinese Olive

The MYB gene family, which plays an important role in regulating gene expression in plants, has been reported in a variety of plants through systematic genome-wide analyses. However, no Chinese olive MYB transcription factors have been reported to date, and the lack of genomic information has led to a lack of functional studies of Chinese olive MYB. In this study, 83 Chinese olive MYB transcription factors were identified based on the combination of the three-generation full-length transcriptome and the second-generation transcriptome (PRJNA749395). Phylogenetic analyses revealed that all four classes of MYB transcription factors were present in Chinese olive fruits and were significantly differentially expressed during developmental periods. The number of the four subfamilies was 54 1R-MYB (MYB-related), 25 2R-MYB (R2R3-MYB), 3 3R-MYB, and 1 4R-MYB, respectively. Twelve classes of R2R3-MYB were differentially expressed in Chinese olive. The R2R3-MYB subfamily plays a central role in the regulation of plant metabolism, particularly in the synthesis of specific classes of phenylpropane compounds, including flavonoids, anthocyanins, and lignans. Consequently, a significant proportion of studies on MYB transcription factors in plants have focused on the R2R3-MYB subfamily [22]. However, the gradual increase in the number of MYB-related genes from bryophytes to flowering plants suggests that MYB-related proteins have evolved diverse functions through extensive expansion during plant evolution [23]. A growing number of studies have also revealed that MYB-related proteins play an important role in the transcriptional regulation of various biological processes, including epidermal cell development, leaf senescence, circadian regulation, photoperiodic regulation, specific protein recognition, DNA-binding proteins, and stress responses, among others [21,23]. In this study, the most differentially expressed members of the MYB-related subfamily were identified in Chinese olives, with a large number of differentially expressed members in the CCA1/R-R-like and TBP-like categories (33 and 16 members, respectively). This suggests that the MYB-related subfamily, especially the CCA1/R-R-like proteins, may play an important role in Chinese olive fruits.

Gene expression and trend analyses have identified a highly expressed MYB transcription factor (*CaMYBR04*, *Isoform0032534*) with an expression pattern consistent with the trend of Chinese olive fruit GA, *β*G, and HTs content. This transcription factor belongs to the CCA1/R-R-like protein in the MYB-related subfamily, which is divided into five groups: TBP-like, TRF-like, I-box-like, CCA1/R-R-like, and CPC-like. The CCA1/R-R-R-like and TBP-like groups are the most numerous. In previous studies, the majority of CCA1/R-R-like genes exhibited a high response at the early stages of plant development [23], a pattern similar to that observed for *CaMYBR04*. This suggests that *CaMYBR04* may play an important role in the early development of Chinese olive fruit. Correlation analysis revealed a highly significant positive correlation between CaMYBR04 expression and GA and *β*G content, with correlation coefficients of 0.832 and 0.909, respectively. This suggests that *CaMYBR04* may regulate *β*G synthesis in Chinese olive fruits and that its relevant functions should be validated.

### 3.2. CaMYBR04 Promotes Fruit-Hydrolysed Tannin Biosynthesis

MYB transcription factors, which typically function as regulators of secondary metabolic pathways in plants, are crucial regulators of the phenylpropanoid metabolic pathway in plants and play a pivotal role in regulating the synthesis of various phenylpropanoid compounds [24,25]. In recent years, the transcription factor PbrMYB24 was identified as a key regulator of lignin and cellulose biosynthesis in pear fruit stone cells [26]. In grapefruit, the transcription factor CgMYB58 was identified to regulate lignin formation, which granulates the juice cells and affects fruit flavour and taste [27]. The majority of MYBs act as positive regulators in flavonoid biosynthesis, enhancing the expression of enzyme genes, while some act as negative regulators, inhibiting the production of anthocyanin [28,29,30]. For example, the Citrus CsMYB3 regulates anthocyanin biosynthesis in citrus by forming an “activator-and-repressor” loop with CsRuby1 [31]. Additionally, MYB can regulate fruit flavonoid composition and quality by binding to other transcription factors to form a complex. In general, MYBs involved in flavonoid biosynthesis can be classified into two categories: stand-alone MYB transcription factors and transcriptional complexes, including the MYB-bHLH-WDR (MBW) complex or the MYB-bHLH complex [32]. For instance, MdMYB305-MdbHLH33-MdMYB10 and MdHY5-MdWRKY41-MdMYB regulate the balance of sugars and anthocyanins and proanthocyanidins in red-fleshed apple fruit [33,34]. The biosynthesis of flavonoid compounds is regulated by the pomegranate MYB5-like and bHLH transcription factors, which promote the production of dihydroflavonols [35]. The DkMYB2, DkMYB4, DkMYC1 (bHLH), and DkWDR1 (WD40) proteins were involved in the process. In a complex of proteins that work together to regulate the expression of proanthocyanidin synthesis genes in persimmon and affect astringency formation [36], the WDR complex also mediates transcriptional regulation of the flavonoid biosynthesis pathway in a variety of berries with high phenolic content [37].

This is despite the fact that the regulation of phenylpropanoid metabolism is more extensively studied in important horticultural species. However, information on the regulation of polyphenol biosynthesis is still limited in many branching pathways compared to the level of knowledge on other plants’ secondary metabolic pathways, especially outside the phenylpropanoid pathway, where studies on the regulation of compounds such as coumarins, quinones, and hydrolysable tannins are still lacking [38]. To date, the hydrolysable tannin biosynthesis pathway has not been well studied, and only a few of these genes have been functionally demonstrated [39,40,41,42]. It has been demonstrated that the metabolic pathways of hydrolysable tannins, anthocyanins, and proanthocyanidins (condensed tannins) share a common origin from the shikimate pathway. Furthermore, it has been demonstrated that the biosynthesis of these compounds may be regulated by similar regulatory elements and may share the same genetic mechanism [43]. This information informs our research into transcription factors regulating Chinese olive hydrolysed tannins. It was found that MYB transcription factors regulate the synthesis of condensed tannins in fruits. The expression level of *MYBPA1* in *Prunus persica* was found to be consistent with the level of condensed tannins, suggesting that *MYBPA1* in *Prunus persica* is a potential transcription factor that regulates its condensed tannins [44]. Furthermore, it was observed that the accumulation of proanthocyanidin in grapes commences at the fruit setting stage and is determined by the presence of *MYBPA1*, *MYBPA2*, and *MYB5a*. The downregulation of genes such as *MYB5a* and *MYBPA2* results in a shift from proanthocyanidin biosynthesis to anthocyanin biosynthesis when the fruit enters the second growth stage [45]. The expression of structural genes involved in the condensed tannin synthesis pathway is driven by the MBW complex. In strawberries, the MBW complex is composed of *FaMYB9/FaMYB11*, *FabHLH3*, and *FaTTG1*, which upregulates the expression of genes encoding anthocyanin synthesis-related genes, thereby increasing strawberry proanthocyanidin levels [46]. Furthermore, FaMYB123 interacts with FabHLH3 to regulate late steps in anthocyanin and flavonol biosynthesis during strawberry fruit ripening [32]. Persimmon *DkMyb4* is involved in proanthocyanidin biosynthesis at an early stage of fruit development, acting as a direct regulator of proanthocyanidin pathway genes. Downregulation of *DkMyb4* resulted in the specific downregulation of proanthocyanidin biosynthesis, which in turn improved proanthocyanidin content and fruit astringency traits in persimmon [47]. However, there is a paucity of literature on the regulation of hydrolysed tannins by MYB transcription factors in plants. With regard to the hydrolysed tannin biosynthesis pathway, it has been demonstrated that Arabidopsis AtMYB15 is a regulator of its upstream shikimate pathway [48]. PgMyb308-like in pomegranate has been shown to increase the levels of shikimic acid, aromatic amino acids, and lignin in pomegranate while inhibiting the synthesis of flavonoids and hydrolysable tannins [49].

Strawberry and *C. album* fruits contain high amounts of bioactive polyphenols, in particular ellagic acid and ellagitannins [12]. They share a similar metabolic pathway and both synthesise *β*G, which represents an important step in the synthesis of hydrolysed tannins [50,51]. Consequently, strawberry is a more suitable model plant for genetic transformation than Arabidopsis. This study found that the Chinese olive *CaMYBR04* gene was highly expressed during Chinese olive fruit development, with a pattern of expression consistent with the trend of Chinese olive fruit GA, *β*G, and HT content. The *CaMYBR04* gene was cloned using full-length CDS and successfully transiently overexpressed in strawberry fruits. The results showed that *CaMYBR04* transformation could enhance the synthesis of hydrolysable tannins by *β*G in fruits and elevate the hydrolysable tannin content of fruits. However, which target genes are specifically regulated by *CaMYBR04*, and there is interaction between them? Is there a complex for more complex regulation? These need to be further studied in the future.

## 4. Materials and Methods

### 4.1. Test Material

#### 4.1.1. Plant Material

The fruits of the *C. album* cultivar ‘Changying’, cultivated at the *C. album* plantation located in Minhou County, Fujian Province, China (26°13′ N, 119°02′ E, 127 m altitude), were used as materials for this study. Chinese olive flesh samples were selected at 20 DAF (days after flowering), 40 DAF, 70 DAF, and 110 DAF, with three biological replicates in each period. The fruits were washed, pitted, and treated with liquid nitrogen and stored at −80 °C in the refrigerator. The total RNA of Chinese olive fruit was employed as the test material for gene cloning.

The subcellular localisation material was *Nicotiana benthamiana*, which was cultivated in an artificial climate chamber with a day/night temperature of 28 °C/22 °C, a light/dark cycle of 16 h/8 h, and a relative humidity of 70%. The plants were cultured for approximately 4–6 weeks for subcellular localisation experiments.

The test material for transient transformation was selected from forest strawberry (*Fragaria vesca*), variety ‘Ruegen’, and the plants were cultivated in an artificial climate chamber under the following conditions: 23 ± 1 °C, light/dark cycle of 16 h/8 h, and relative humidity of 70%. Strawberry fruits at the small green fruit stage (10–12 days after flowering) with uniform growth and no pests or diseases were selected for transient overexpression.

#### 4.1.2. Strains and Vectors

Strains: *E. coli* Trans1-T1 (TransGene, Beijing, China), Agrobacterium tumefaciens GV3101 (Shanghai Vidi, Shanghai, China), Expression competent cell BL21 (DE3) (TransGen).

Vectors: pENTRTM/D-TOPO vector (Invitrogen, Waltham, MA, USA), pK7FWG2, pET28a.

### 4.2. Identification and Analysis of the Chinese Olive MYB Transcription Factor

#### 4.2.1. Identification of MYB Transcription Factors and Analysis of Protein Physicochemical Properties

The MYB transcription factors identified in the Chinese olive PicBio SMRT-seq database were collected and the RNA-seq data were used to obtain differentially expressed MYB protein sequences (PRJNA749395). Each protein sequence was submitted to the HMMER online software (http://www.ebi.ac.uk/Tools/hmmer/, accessed on 1 September 2019) to confirm the presence of the MYB structural domain (PF00249), thereby identifying the Chinese olive MYB transcription factor family members. The protein physicochemical properties, including molecular weight and isoelectric point, were analysed for the Chinese olive MYB transcription factor members using the Expasy. The subcellular localisation of the CaMYBs was predicted using the CELLO v.2.5 online website.

#### 4.2.2. Phylogenetic Tree Construction

A phylogenetic tree was constructed using identified Chinese olive MYB transcription factor members and Arabidopsis MYB protein sequences to analyse the classification and phylogeny of Chinese olive MYB transcription factors. Arabidopsis MYB protein sequences were obtained from The Arabidopsis Information Resource (TAIR, https://www.arabidopsis.org/browse/genefamily/index.jsp, accessed on 1 September 2019) and included 133 MYB and 68 MYB-related members [18,23]. A phylogenetic tree was constructed using the NJ method with a bootstrap value of 1000.

#### 4.2.3. Conservative Motif Identification

The MEME program was used to identify the conserved motifs of Chinese olive MYB transcription factors, with up to 20 motifs per sequence. The phylogenetic trees and motifs of Chinese olive MYB transcription factor members were rearranged using the TBtools software (http://www.tbtools.com, accessed on 1 April 2020).

#### 4.2.4. Expression Pattern Analysis of Candidate CaMYBs

The expression patterns of Chinese olive MYB transcription factor members were analysed based on RNA-seq data at four periods. Candidate genes were screened based on trend analysis to further screen for potential key MYB transcription factors regulating Chinese olive *β*G formation. Spearman’s correlation analysis was conducted using SPSS 19.0 software to examine the relationship between the expression of candidate CaMYB transcription factors and the GA and *β*G content of Chinese olive fruits. The results indicated a statistically significant correlation (*p* < 0.001).

### 4.3. Cloning and Overexpression Vector Construction of the CaMYBR04 Transcription Factor

#### 4.3.1. Total RNA Extraction of Chinese Olive Fruit and cDNA Synthesis

The Chinese olive pulp was ground well with liquid nitrogen and the total RNA was extracted from the fruit using the instructions provided with the polysaccharide and polyphenol extraction kit (TIANGEN, Beijing, China). The quality and concentration of the extracted RNA were then analysed by 1.2% agarose gel electrophoresis with a miniature ultraviolet spectrophotometer Nanodrop 2000 (Thermo Scientific, Waltham, MA, USA). The synthesis of the first strand of cDNA was conducted using total RNA extracted from Chinese olive fruit as a template, following the protocol outlined in the TIANGEN^®^ FastKing DNA Dispelling RT SuperMix method. The resulting cDNA was purified and stored at −20 °C for subsequent use as a template for gene cloning.

#### 4.3.2. Cloning and Overexpression Vector Construction of CaMYBR04

The cDNA of Chinese olive fruit was used as a template for PCR amplification of the CDS fragment of *CaMYBR04*, a key MYB transcription factor in Chinese olive. The primer sequences are shown in Table 1, and the lowercase letters indicate the junction sequences for ligating TOPO vectors. The sequencing results of the positive clones were analysed, and the correct *CaMYBR04* positive plasmid was homologously replaced with the overexpression vector pK7FWG2 by LR reaction to obtain the recombinant plasmid pK7FWG2-*CaMYBR04*. This was stored at −20 °C for spare parts.

### 4.4. Subcellular Localisation

Under the methodology outlined in the Liu et al. study [52], the plasmids pK7FWG2-*CaMYBR04* and pK7FWG2 were transformed into Agrobacterium competent cells GV3101 via a freeze–thawing process. Following positive identification, the correct bacterial solution was glycerol-preserved and stored at −80 °C for future use.

Once the tobacco plants had been cultivated for approximately four to six weeks, the Agrobacterium solution containing the pK7FWG2-*CaMYBR04* and the empty plasmid pK7FWG2, stored at −80 °C, was taken for activation. This was achieved by resuspending the bacteria until the OD600 reached 0.6–0.8. Subsequently, the Agrobacterium was transiently transformed on the abaxial side of tobacco leaves to observe the subcellular localisation of the introduced genetic material.

### 4.5. Eukaryotic Heterologous Transient Overexpression

Ruegen strawberry fruits were used as test material for transient overexpression of the Chinese olive *CaMYBR04* transcription factor. By the methodology outlined by Guadalupe et al. [53] and Zhao et al. [54], the activated Agrobacterium solution containing pK7FWG2-*CaMYBR04* and the empty plasmid pK7FWG2 was transformed into strawberry fruits at the stage of small green fruits [50]. Strawberry fruits were collected at 5 d post-injection, with three biological replicates per treatment containing at least 10 fruits per biological replicate. They were immediately treated with liquid nitrogen and stored in a −80 °C refrigerator. Each transiently transformed strawberry fruit was positively identified, and gene expression was detected by RT-qPCR, with the DNA-binding protein (DBP) gene serving as an internal reference [55]. The primer sequences are shown in Table 2. After this, total protein was extracted for Western blot detection.

#### 4.5.1. Western Blot Assay

##### Protein Extraction

Once the positive strawberry fruit had been fully ground with liquid nitrogen, 100 mg of powder was transferred to a 1.5 mL centrifuge tube. Subsequently, 100 μL of protein extract 2× Sample Buffer was added, and the mixture was vortexed and shaken until fully mixed. The samples were immersed in a metal bath at 100 °C for 10 min and then centrifuged at 12,000 rpm for 5 min. Thereafter, 10 μL of the supernatant was pipetted onto an SDS-PAGE gel to separate the proteins.

##### Transferred Membrane

The separated gel, after electrophoresis, was removed and placed in 1× Transfer Buffer together with the NC membrane. Subsequently, the membrane was transferred to a TRANS-BLOT^®^ SD SEMI-DRY TRANSFER CELL (Bio-Rad, Hercules, CA, USA) for a period of 70 min at 10 V.

##### Enclosure, Antibody Incubation

The enclosure treatment involved the use of 5% non-fat dry milk (20 mL PBST + 1 g milk) for a period of 3 h at a speed of 50 rpm/min on a horizontal shaker. A volume of 3 μL of the primary antibody (Anti-GFP Mouse Monoclonal Antibody) (TransGen) was added to 20 mL of 5% milk, and the mixture was incubated overnight at 4 °C. Following this, the samples were washed 5 times with PBST for 5 min each. Next, 3 μL of the secondary antibody (Goat Anti-Mouse IgG (H + L), HRP Conjugate) (TransGen) was added to 20 mL of 5% milk and incubated on a shaker for 1 h at room temperature. Then, samples were washed 5 times with PBST for 5 min each.

##### Expose

We took 1 mL of each of the developer solutions A and B (eECL Western Blot Kit, CWBIO, Cambridge, MA, USA) and mixed them well. The NC membrane was immersed in the development mixture, developed for 5 min, and placed in the imager for scanning and imaging.

#### 4.5.2. UPLC-MS/MS Determination of Metabolic Components

Strawberry fruits that tested positive by PCR were determined for GA, *β*G and HTs in strawberry fruits using a method previously described [51].

## 5. Conclusions

Based on the identification and screening of differentially expressed MYB transcription factors in Chinese olive, a total of 83 CaMYBs were obtained. Phylogenetic analyses revealed that all four subfamilies of Chinese olive MYB transcription factors exhibited differential expression. These included 54 1R-MYBs (MYB-related), 25 2R-MYBs (R2R3-MYBs), 3 3R-MYBs, and 1 4R-MYB. The R2R3-MYB subfamily exhibited differential expression in 12 categories, while the MYB-related subfamily displayed differential expression in two categories, CCA1/R-R-like and TBP-like, with 33 and 16 members, respectively. A transcription factor, *CaMYBR04* (*Isoform0032534*), which is highly expressed and highly related to Chinese olive *β*G, was identified based on expression pattern and trend analysis. Heterologous transient transformation of strawberries demonstrated that *CaMYBR04* regulates the further transformation of *β*G in strawberry fruit and increases the HT content of the fruit. The results provided a foundation for further studies on the regulation of the biosynthesis of Chinese olive fruit *β*G and its derivatives.

## Figures and Tables

**Table 1:** Sequences of primers for amplification of *CaMYBR04*.

Gene	Primers	Primer Sequence (5′-3′)
*CaMYBR04* (*Isoform0032534*)	TOPO-F	caccATGCTTTTCGGAGTGAGAGTGGTTGTGG
	TOPO-R	AGCAACACTGATGATGTTGCTGCTGCCCT

**Table 2:** Primer sequences for RT-qPCR of transiently transformed strawberry fruits.

Primers	Primer Sequence (5′-3′)
pK7-gfp-F	ATCATGGCCGACAAGCAGAA
pK7-gfp-R	TCTCGTTGGGGTCTTTGCTC
DBP-F	GGCATCGGAGATGGTACTGT
DBP-R	CCAGCATTCCGAACTTCTTT

## Data Availability

PicBio SMRT-seq and RNA-seq data have been deposited into the NCBI Sequence Read Archive database (PRJNA749395). The data supporting the results of this study are included in the present article and Supplementary Materials.

## Supplementary Materials

The following supporting information can be downloaded at the following link: https://www.mdpi.com/article/10.3390/plants13131837/s1, Table S1: Physicochemical properties and subcellular localization prediction of differentially expressed MYB transcription factors in Chinese olive. Figure S1: Conserved motifs from 83 CaMYBs. Figure S2: Overexpression vector pK7FWG2-CaMYBR04 positive clone detection. Figure S3: PCR identification of partial strawberry fruits transiently transformed by *CaMYBR04.*
