# Supplementary material for: Identification of MYB Transcription Factor, a Regulator Related to Hydrolysable Tannin Synthesis in Canarium album L., and Functional Analysis of CaMYBR04
Source: Plants (Basel). 2024 Jul 4;13(13):1837. doi: 10.3390/plants13131837 (PMC11244293; doi:10.3390/plants13131837)
Supplement: Supplementary file 1 [file plants-13-01837-s001.zip › plants-3018846-supplementary.pdf]

Supplementary Table and Figures

Table S1. Physicochemical properties and subcellular localization prediction of differentially expressed MYB transcription factors in Chinese olive.

NO.	Gene ID	Amino Acids (aa)	Molecular Weight (Da)	Isoelectric Point (pI)	Predicted Subcellular localization
1	Isoform0004472	125	14439.61	9.77	Nuclear/Mitochondrial
2	Isoform0005912	73	8266.94	4.4	Nuclear
3	Isoform0007650	255	29143.8	5.56	Nuclear
4	Isoform0008205	186	20956.88	10.12	Nuclear
5	Isoform0008464	213	23475.59	9.37	Nuclear
6	Isoform0012115	248	27255.43	7.52	Nuclear
7	Isoform0012686	306	33094.32	8.78	Nuclear
8	Isoform0012864	355	38543.94	6.84	Nuclear
9	Isoform0013132	322	36742.27	4.78	Nuclear
10	Isoform0013800	307	33461.88	8.98	Nuclear
11	Isoform0013828	334	37313.46	5.95	Nuclear
12	Isoform0013930	122	13692.41	10.53	Nuclear/Mitochondrial
13	Isoform0014048	318	36491.49	6.23	Nuclear
14	Isoform0014103	240	26230.34	6.67	Nuclear
15	Isoform0015337	352	38223.91	7.7	Nuclear
16	Isoform0015549	310	33526.42	9.11	Nuclear
17	Isoform0016337	303	34275.12	6.47	Nuclear
18	Isoform0017792	115	13223	10.29	Nuclear
19	Isoform0018829	282	30941.08	8.46	Nuclear/Mitochondrial
20	Isoform0019056	263	28953.28	6.9	Nuclear
21	Isoform0019155	284	32547.04	5.74	Nuclear
22	Isoform0019520	326	35586.69	6.02	Nuclear
23	Isoform0020959	294	31428.82	7.25	Nuclear
24	Isoform0022409	273	30707.1	9.28	Nuclear
25	Isoform0022606	310	34545.21	5.53	Nuclear
26	Isoform0022813	288	31584.32	5.87	Nuclear
27	Isoform0023787	263	28938.48	9.27	Nuclear
28	Isoform0023811	307	34021.02	8.21	Nuclear
29	Isoform0025445	258	28813.74	8.89	Nuclear
30	Isoform0026582	254	28169.35	7.97	Nuclear
31	Isoform0026679	284	32579	5.58	Nuclear
32	Isoform0026990	169	19184.86	9.6	Nuclear
33	Isoform0027576	332	36926.55	6.45	Nuclear
34	Isoform0028391	300	33341.17	6.86	Nuclear
35	Isoform0029170	234	25636.72	8.57	Nuclear

36	Isoform0030080	325	36120.26	6.26	Nuclear
37	Isoform0030613	254	27926.24	6.5	Nuclear
38	Isoform0031433	391	43791.62	8.73	Nuclear
39	Isoform0032534	274	30228.94	8.9	Nuclear
40	Isoform0032600	296	32952.7	7.67	Nuclear
41	Isoform0033430	218	24549.42	7.3	Nuclear
42	Isoform0039629	238	26186.73	4.65	Nuclear/Chloroplast
43	Isoform0039652	302	34118.29	5.87	Nuclear
44	Isoform0041032	759	83325.22	6.04	Nuclear
45	Isoform0041142	517	57069.11	8.29	Nuclear
46	Isoform0041598	757	82903.68	6.1	Nuclear
47	Isoform0048595	798	86572.18	5.78	Nuclear
48	Isoform0049183	446	49430.56	8.41	Nuclear
49	Isoform0050789	459	50843.52	6.89	Nuclear
50	Isoform0053058	538	61651.34	9.52	Nuclear
51	Isoform0054809	752	82552.24	6.02	Nuclear
52	Isoform0059160	224	24570.52	9.21	Nuclear
53	Isoform0060460	759	83244.94	5.96	Nuclear
54	Isoform0061174	568	64080.45	6.48	Nuclear
55	Isoform0061481	787	85242.48	5.39	Nuclear
56	Isoform0061803	184	21856.87	9.18	Nuclear
57	Isoform0064116	257	28725.59	9.35	Nuclear
58	Isoform0070309	488	53912.33	5.92	Nuclear
59	Isoform0072917	1048	115485.8	5.32	Nuclear
60	Isoform0073568	651	71310.9	6.08	Nuclear
61	Isoform0075431	248	27317.63	5.33	Nuclear
62	Isoform0077070	1044	115092.8	5.07	Nuclear
63	Isoform0078904	776	85727.64	6.65	Nuclear
64	Isoform0096858	1271	139348.7	5.73	Nuclear
65	Isoform0099167	327	35403.24	8.3	Nuclear
66	Isoform0104550	1369	149768.5	5.8	Nuclear
67	Isoform0107496	1390	152318.3	5.55	Nuclear
68	Isoform0109568	1766	193112.1	6.07	Nuclear
69	Isoform0110703	1634	178418.8	5.73	Nuclear
70	Isoform0112764	1696	184741.8	5.9	Nuclear
71	Isoform0112861	111	11822.22	10.73	Nuclear
72	Isoform0113197	1758	192645.3	5.96	Nuclear
73	Isoform0116236	207	22873.92	9.1	Nuclear
74	Isoform0117645	263	29573.56	8.89	Nuclear
75	Isoform0118065	359	40513.05	5.29	Nuclear
76	Isoform0118249	309	34966.07	7.77	Nuclear
77	Isoform0119010	273	30862.49	6.26	Nuclear
78	Isoform0119674	291	32700.96	7.99	Nuclear
79	Isoform0120030	530	59857.87	6.2	Nuclear

80	Isoform0120049	238	26217.76	5.23	Nuclear
81	Isoform0120083	262	29797.15	6.43	Nuclear
82	Isoform0120575	345	39144.39	6.47	Nuclear
83	Isoform0122550	71	8339.57	11.77	Nuclear/Mitochondrial

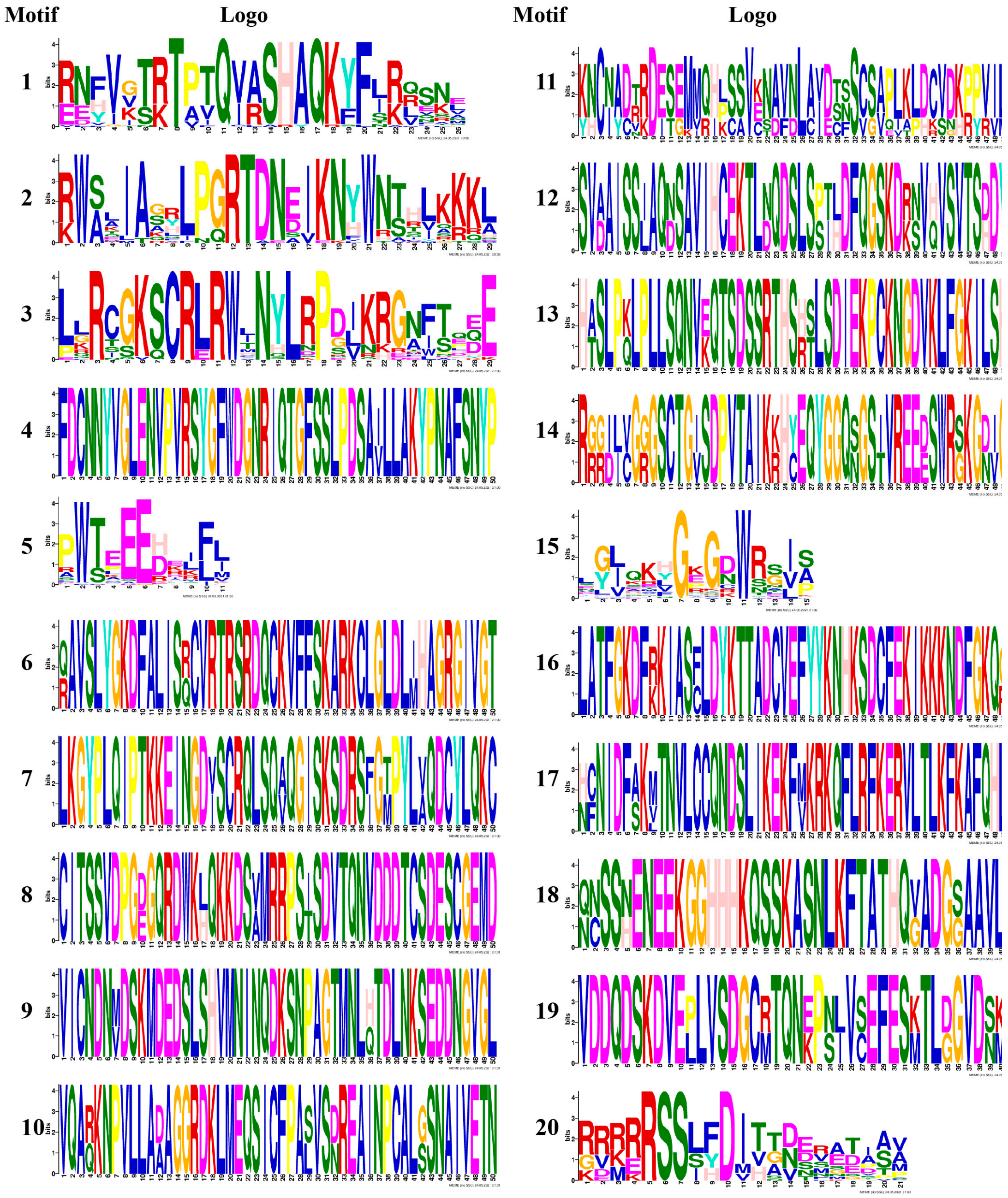

Figure S1. Conserved motifs from 83 CaMYBs.

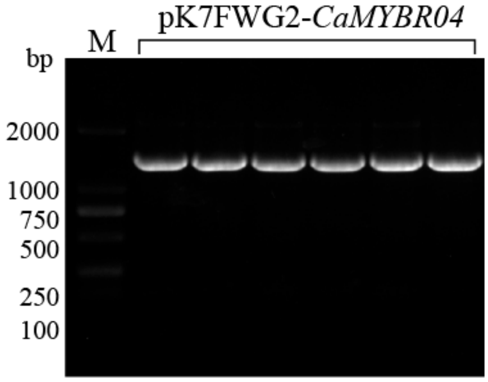

Figure S2. Overexpression vector pK7FWG2-CaMYBR04 positive clone detection.

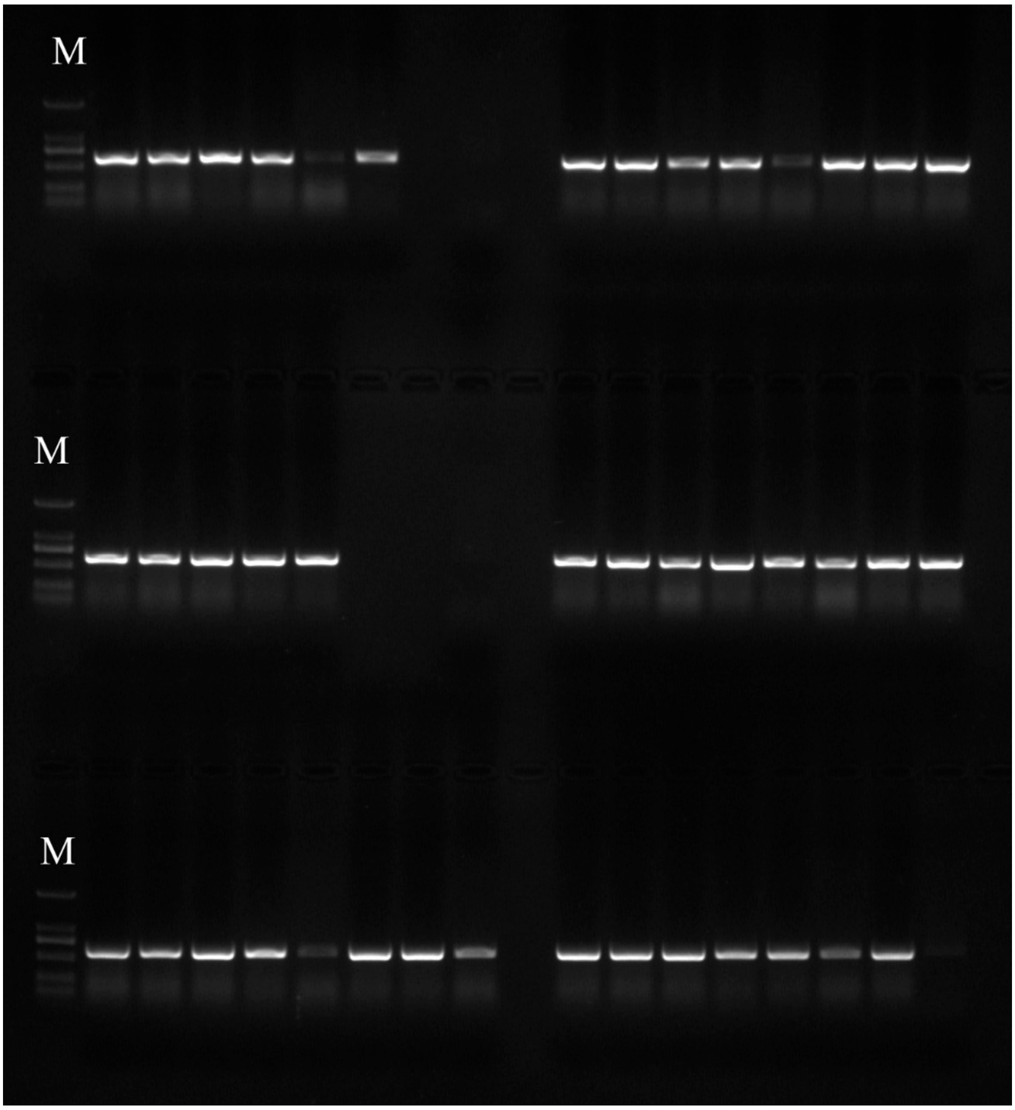

Figure S3. PCR identification of partial strawberry fruits transiently transformed by *CaMYBR04.* M: DNA Marker DL2000; The underlined lanes are CK samples, the others are strawberry samples transiently transformed by *CaMYBR04*.
